# Subjective cognitive complaints and blood biomarkers of neurodegenerative diseases: a longitudinal cohort study

**DOI:** 10.1186/s13195-023-01341-3

**Published:** 2023-11-11

**Authors:** Laura Perna, Hannah Stocker, Lena Burow, Léon Beyer, Kira Trares, Carolin Kurz, Selim Gürsel, Bernd Holleczek, Maia Tatò, Konrad Beyreuther, Ute Mons, Klaus Gerwert, Robert Perneczky, Ben Schöttker, Hermann Brenner

**Affiliations:** 1 Department Genes and Environment, Max Planck Institute of Psychiatry, 80804 Munich, Germany; 2 Division of Mental Health of Older Adults, Department of Psychiatry and Psychotherapy, University Hospital, LMU, Munich, Germany; 3 Division of Clinical Epidemiology and Aging Research, German Cancer Research Center (DKFZ), Heidelberg, Germany; 4 Network Aging Research (NAR), Heidelberg University, Heidelberg, Germany; 5 Faculty of Biology and Biotechnology, Department of Biophysics, Ruhr-University Bochum, 44801 Bochum, Germany; 6 Center for Protein Diagnostics (ProDi), Ruhr-University Bochum, 44801 Bochum, Germany; 7 Saarland Cancer Registry, 66117 Saarbrücken, Germany; 8 Department of Cardiology, Faculty of Medicine, University Hospital Cologne, University of Cologne, Cologne, Germany; 9 Ageing Epidemiology (AGE) Research Unit, School of Public Health, Imperial College London, London, UK; 10 German Center for Neurodegenerative Diseases (DZNE), Munich, Germany; 11 Munich Cluster for Systems Neurology (SyNergy), Munich, Germany; 12 Sheffield Institute for Translational Neurology (SITraN), University of Sheffield, Sheffield, UK

**Keywords:** Subjective cognitive decline, Dementia, Depression, Glial fibrillary acidic protein, *APOE* ε4, Older people

## Abstract

**Background:**

Subjective cognitive complaints (SCC) have been mostly studied in the context of Alzheimer’s disease in memory clinic settings. The potential of combining SCC with genetic information and blood biomarkers of neurodegenerative diseases for risk assessment of dementia and depression in the absence of dementia among community-dwelling older adults has so far not been explored.

**Methods:**

Data were based on a population-based cohort of 6357 participants with a 17-year follow-up (ESTHER study) and a clinic-based cohort of 422 patients. Participants of both cohorts were grouped according to the diagnosis of dementia (yes/no) and the diagnosis of depression in the absence of dementia (yes/no). Participants without dementia included both cognitively unimpaired participants and cognitively impaired participants. Genetic information (*APOE* ε4 genotype) and blood-based biomarkers of neurodegenerative diseases (glial fibrillary acidic protein; GFAP, neurofilament light chain; NfL, phosphorylated tau181; p-tau181) were available in the ESTHER study and were determined with Simoa Technology in a nested case–control design. Logistic regression models adjusted for relevant confounders were run for the outcomes of all-cause dementia and depression in the absence of dementia.

**Results:**

The results showed that persistent SCC were associated both with increased risk of all-cause dementia and of depression without dementia, independently of the diagnostic setting. However, the results for the ESTHER study also showed that the combination of subjective complaints with *APOE* ε4 and with increased GFAP concentrations in the blood yielded a substantially increased risk of all-cause dementia (OR 5.35; 95%CI 3.25–8.81, *p*-value < 0.0001 and OR 7.52; 95%CI 2.79–20.29, *p*-value < 0.0001, respectively) but not of depression. Associations of NfL and p-tau181 with risk of all-cause dementia and depression were not statistically significant, either alone or in combination with SCC, but increased concentrations of p-tau181 seemed to be associated with an increased risk for depression.

**Conclusion:**

In community and clinical settings, SCC predict both dementia and depression in the absence of dementia. The addition of GFAP could differentiate between the risk of all-cause dementia and the risk of depression among individuals without dementia.

**Supplementary Information:**

The online version contains supplementary material available at 10.1186/s13195-023-01341-3.

## Background

Subjective cognitive decline (SCD) is defined as self-experienced cognitive deterioration in the absence of objective evidence of impairment [1], occurring mostly in the context of Alzheimer’s disease [2]. SCD is associated with an increased risk of progression to mild cognitive impairment (MCI) and dementia [3–6] and with biomarkers of neurodegenerative diseases [7–9]. In memory clinic cohorts, patients with SCD and evidence of biomarker pathology according to the ATN classification system [10] show an accelerated longitudinal cognitive decline and an increased risk of developing dementia [7, 11].

The assessment of SCD as defined by international criteria [1, 2] requires elaborate diagnostic procedures including comprehensive neuropsychological tests and brain imaging. Hence, this pathology has been mostly studied in the context of memory clinic settings. However, in community-based cohorts, the prevalence of self-experienced cognitive decline is widespread even among younger participants [12–15], including the age group 30–39 years [13], and large studies point to a prevalence of approximately 50–60% among older persons [12, 13, 15]. Thus, it is of high relevance to explore the specific diagnostic value of self-experienced cognitive decline reported in community settings, irrespective of comprehensive neuropsychological examinations. In this work, we define this condition as subjective cognitive complaints (SCC). This definition focuses on the subjective experience of cognitive decline and it does not rule out that specific diagnostic procedures not commonly available in community settings might find objective evidence of cognitive impairment or pathological levels of biomarkers of neurodegenerative diseases.

Both in clinical and community settings self-reported cognitive decline alone is of limited diagnostic utility because it is nonspecific and common to several medical conditions [2]. The question then arises whether exploring possible biological underpinnings of SCC and, specifically, combining SCC with genetic and biomarker information could have any value for risk assessment and for differentiating between SCC leading to risk of all-cause dementia or to risk of depression in the absence of dementia. Such conditions are challenging because early-stage dementia and depression among older people have overlapping signs and symptoms [16]. However, an early differential risk assessment would be of high clinical relevance both for supporting clinicians in decisions relevant to treatment, including choice of medication use, and for helping patients in making decisions relevant to lifestyle changes and plans for the future.

The main aim of this study was to assess whether combining SCC with blood biomarkers of neurodegenerative diseases (glial fibrillary acidic protein (GFAP), neurofilament light chain (NfL), and phosphorylated tau181 (p-tau181)) and information on apolipoprotein E (*APOE*) ε4 increases the predictive value of SCC for risk of all-cause dementia among community-dwelling older adults and whether it contributes to the differentiation between the risk of all-cause dementia and the risk of a first depressive episode in the absence of dementia. A secondary aim was to investigate whether the possible diagnostic value of SCC not substantiated by biological information differs depending on recruitment settings such as community- or clinic-based settings [17].

## Methods

### Study population

#### Population-based data

The data for the main goal of the study are based on a community-based prospective cohort of older adults (*n* = 9940) followed up for 17 years (the ESTHER study). ESTHER participants were recruited in 2000–2002 through general practitioners (GPs). The eligibility criteria were age between 50 and 75 years, sufficient knowledge of the German language, residence in the German state of Saarland, and willingness to attend a general health examination performed by GPs. To not impair the generalizability of the study, no specific exclusion criterium based upon cognitive functioning was applied [18, 19]. Blood samples collected at baseline were stored at − -80 °C. Follow-up measurements were conducted 2, 5, 8, 11, 14, and 17 years after baseline. All participants filled in a health questionnaire and GPs provided medical records.

**Fig. 1:**
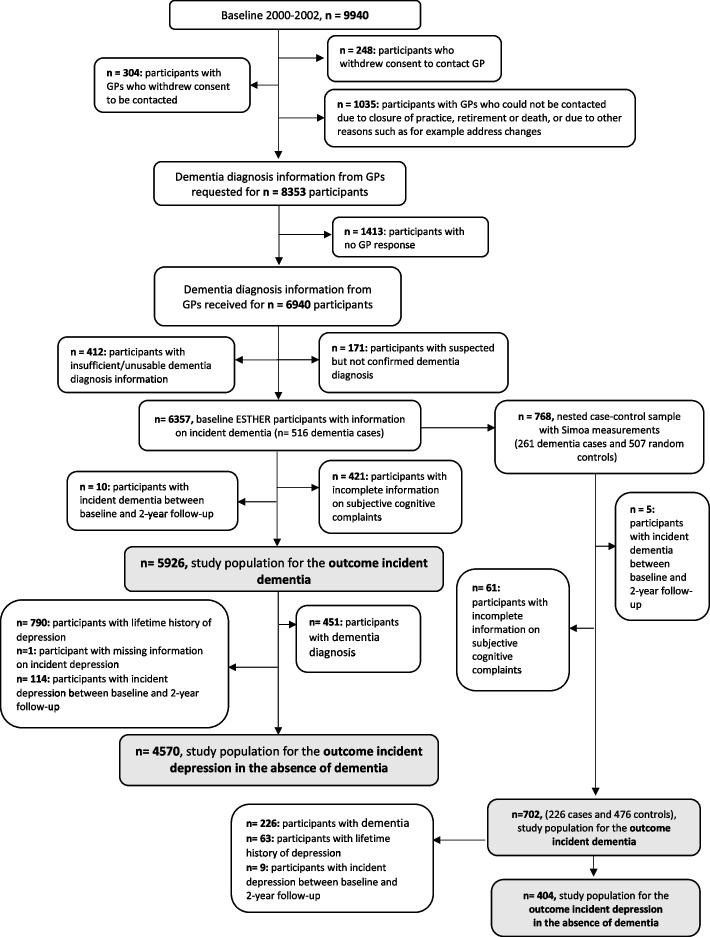
Flow chart of the ESTHER study

During the 14- and 17-year follow-up, the GPs of all ESTHER study participants, including those of participants who had dropped out of the study or had died, were contacted and were asked to provide information relating to a diagnosis of dementia since enrollment and to send the corresponding medical records, if available. In total, GPs of *n* = 6357 participants provided usable dementia diagnosis information (Fig. 1). For the purpose of this study, participants with incomplete information relating to SCC (*n* = 421) and with a dementia diagnosis between recruitment and 2-year follow-up measurement were excluded. Specifically, in order to minimize the possibility of undiagnosed dementia at baseline, we excluded all participants with a diagnosis of dementia dated up to 3 years after recruitment (*n* = 10), so that *n* = 5926 ESTHER participants remained for the analyses relating to the outcome all-cause dementia. For the outcome relating to the risk of a first depressive episode in the absence of dementia participants with an incident diagnosis of dementia over all follow-up measurements were additionally excluded (*n* = 451) , because these analyses aimed to disentangle depression from all-cause dementia. Furthermore, participants with incident depression between baseline and the 2-year follow-up (*n* = 114), with a lifetime history of depression (*n* = 790), and with missing information on incident depression (*n* = 1) were also excluded, reducing the final sample to *n* = 4570 for the outcome depression in the absence of dementia.

#### Clinic-based data

The data relating to the secondary aim of the study dealing with the comparability of the informative value of SCC in community and clinical settings are cross-sectional, and they were collected within the memory clinic embedded in the Alzheimer Treatment and Research Center (ATFZ) at the Department of Psychiatry and Psychotherapy of the University Hospital of Munich (LMU) between August 2020 and November 2022. The eligibility criteria were age ≥ 60 years, a voluntary appointment with the memory clinic in order to receive dementia diagnostics, and the capacity to give informed consent. The exclusion criteria were illiteracy, insufficient knowledge of the German language, and inability to fill in health questionnaires due to cognitive deterioration.

All patients contacting the memory clinic for dementia diagnostics and fulfilling the inclusion criteria were included in this study. During the recruitment time, all ATFZ patients, in addition to receiving standardized procedures for dementia diagnostics, were asked to complete a comprehensive health questionnaire, which was sent via mail, including exactly the same subjective cognitive questions included in the health questionnaire administered to the ESTHER participants. In total, *n* = 430 patients ≥ 60 years were recruited.

### Assessment of dementia

Dementia diagnoses in the ESTHER cohort were made in community settings with heterogeneous diagnostic procedures, and the most common diagnoses were Alzheimer’s dementia (*n* = 165) and vascular dementia (*n* = 200). However, according to the available medical records, cerebrovascular pathologies (especially vascular encephalopathy, cerebral infarction, microangiopathy) were highly prevalent among dementia cases, including those cases reported as Alzheimer’s dementia. As explained in more detail in a previous work [20], we hence assume that in the ESTHER cohort, independently of the specific dementia type reported as the primary diagnosis, mixed pathologies were most likely to be present in the great majority of dementia cases, and we therefore grouped all dementia diagnoses in one category called “all-cause dementia.”

In the memory clinic, dementia diagnostic procedures included neuropsychological assessment based on the Consortium to Establish a Registry for Alzheimer’s disease – neuropsychological assessment battery plus (CERAD-plus); neurological, physical, and psychiatric examinations; blood tests; electroencephalogram; brain structural MRI scans; and, if appropriate, a lumbar puncture for markers of neurodegeneration. Dementia diagnoses included Alzheimer’s disease, vascular dementia, mixed dementia, Lewy body dementia, Parkinson’s disease dementia, and unspecified dementia. In order to enhance comparability with the ESTHER sample, we grouped all dementia forms reported in the memory clinic in one category (dementia yes/no).

### Assessment of depression

Both in the ESTHER study and in the memory clinic sample, diagnoses of depression were principally made according to the International Classification of Diseases (ICD-10), because this is the official classification for the encoding of medical diagnoses in Germany.

In the ESTHER study, diagnoses of depression were made in community settings by different medical doctors according to the ICD-10 classification, and they were mostly based on clinical observations involving heterogeneous procedures. The outcome depression indicated the first depressive episode occurring during the follow-up duration of the ESTHER study. Specifically, it indicated a medical diagnosis collected at the 2-, 5, 8-,11-, 14-, and 17-year follow-up among participants without a lifetime history of depression. The medical diagnoses were self-reported and collected through health questionnaires administered to participants.

In the memory clinic sample, diagnoses of depression were made following standardized psychiatric and neurological examinations. The 15-item Geriatric Depression Scale (GDS) was routinely administered as a possible support to clinical observations with a value ≥ 5 indicating depressive symptoms. Among participants of the memory clinic, the diagnosis of depression points either to a first depressive episode or to recurrent depression.

### Assessment of SCC

In the self-administered health questionnaire of the ESTHER study at baseline, the following question relating to short-term memory was asked: “Do you have difficulty remembering things that have happened in the recent past (hours to a few days? yes/no).” In the 2-year follow-up, this memory question was replaced by three more specific cognitive questions: “Please, mark if the following statements apply to you just sometimes, always, or never. (1) Lately, I confuse names, phone numbers, or dates; (2) Lately, I misplace things; (3) Lately, I forget names and numbers.” All three cognitive questions were coded with a score ranging from 0 to 2 (0 = never, 1 = sometimes, 2 = always) so that the total score ranged from 0 to 6 with 0 indicating no complaints at all and 6 indicating very frequent complaints in all cognitive questions. To increase the reliability of the self-reported complaints, a combination of the baseline question relating to the short-term memory and of the 2-year follow-up total score was used. The cognitive groups were categorized as follows: (1) no SCC—participants with 0 points in the total score and no short-term memory difficulties (reference group); (2) occasional SCC—participants with > 0 and ≤ 2 points in the total score independently of short-term memory difficulties; and (3) persistent SCC—participants with ≥ 3 points in the total cognitive score and short-term memory difficulties.

The self-administered health questionnaire of the memory clinic included exactly the same three cognitive questions as those included in the ESTHER questionnaire described above. The question relating to the short-term memory was either reported directly to the study doctor or in the health questionnaire, and it was reported at the same time point as the three cognitive questions. The scoring system was the same as the one used for the ESTHER cohort.

### Laboratory measurements

#### Blood biomarkers of neurodegenerative diseases

Concentrations of GFAP, NfL, and p-tau181 in the blood were measured in a subgroup of the ESTHER cohort in a nested case–control design consisting of 261 all-cause dementia cases and 507 controls [20]. The measurements were performed in a single batch in lithium-heparin plasma of baseline samples at the Center for Protein Diagnostics (PRODI) of the Ruhr-University Bochum (Germany) using the single molecule array (SIMOA) Neurology 4-Plex E Advantage Kit and pTau-181 Advantage V2 Kit (Quanterix, USA) on a HD-X Analyzer as per the manufacturer’s instructions [21]. Measurements of amyloid beta could not be used due to its low levels in lithium-heparin plasma [22]. After excluding participants with missing information relating to SCC (*n* = 61) and dementia development up to 3 years from recruitment (*n* = 5), *n* = 702 participants remained for analyses with biomarkers (Fig. 1).

### *APOE* ε4 genotyping

*APOE* ε4 genotype was determined based on allelic combinations of single nucleotide polymorphisms (SNP) rs7412 and rs429358 using TaqMan SNP genotyping assays. Genotypes were analyzed in an endpoint allelic discrimination read using a PRISM 7000 Sequence detection system (Applied Biosystems) [20].

### Other variables

Both in the population and in the memory clinic setting, sociodemographic and health variables were included in the participants’ questionnaire and partially collected or validated through available medical records. In the clinic setting, such data were additionally collected by study doctors during the anamnesis.

### Statistical analyses

Descriptive statistics were used to show the baseline characteristics of the cohorts.

Multivariable logistic regression models were run to assess associations of SCC, *APOE* ε4, and markers of neurodegenerative diseases alone or in combination with risk of all-cause dementia and risk of depression in the absence of dementia. Logistic regression models were adjusted for age (continuous), sex, educational level, lifetime history of stroke, myocardial infarction, diabetes, lifetime history of depression, and *APOE* ε4 (except in models including *APOE* as the independent variable of interest). In the clinic-based sample, the reference category for the logistic regression models included both the absence of SCC and occasional SCC because patients with no SCC were too few. The models performed with clinical data were adjusted for age (continuous), sex, and educational level (continuous). Biomarker values were divided in the respective study populations into quartiles (*Q*), and the highest quartile (*Q*_4_) was compared with the other three quartiles (*Q*_1–3_), which served as the reference group. Participants carrying the *APOE* ε4 allele (ε2ε4, ε3ε4, ε4ε4) were classified as *APOE* ε4 + and those not carrying the ε4 allele (ε2ε2, ε3ε2, ε3ε3) as *APOE* ε4 − . The results were presented as odds ratios (OR) and 95% confidence intervals (CI). For the cross-sectional data collected in the memory clinic setting, we performed additional sensitivity analyses excluding participants with mild cognitive impairment (MCI). The statistical software SAS, version 9.4, Cary, NC, USA, was used for all data.

## Results

### Community-based cohort

In the ESTHER cohort, there were more women than men (54% and 46%, respectively; Table 1), and the great majority of participants were younger than 65 years (70%) at baseline and had low educational level (≤ 9 years of school education; 71%). The mean years from baseline recruitment until the event (dementia diagnosis) among cases was approximately 11 years in the total sample and 10 years in the sample with SIMOA measurements. Given the long follow-up time until the event, we can assume that most participants with dementia diagnosis were either cognitively unimpaired or only mildly cognitively impaired at recruitment.

**Table 1:** Baseline characteristics of ESTHER participants with information on dementia diagnosis and biomarkers of neurodegenerative diseases

	**Baseline (2000–2002)**	**Subgroup with biomarker measurements**		***p*-value**^**b**^
	**Study sample (*n* = 5926), *N* (%)**	**Cases**^**a**^** (*n* = 226), *N* (%)**	**Controls**^**a**^** (*n* = 476), *N* (%)**	
**Sex**				
Female	3224 (54.4)	127 (56.2)	259 (54.4)	
Male	2702 (45.6)	99 (43.8)	217 (45.6)	0.6573
**Education**				
≤ 9 years	4219 (71.2)	187 (82.7)	367(77.1)	
10–11 years	868 (14.6)	16 (7.1)	49 (10.3)	
≥ 12 years	719 (12.1)	15 (6.6)	51 (10.7)	0.0810
***APOE*** **ε4 genotype**				
*APOE* ε4 + ^c^	1466 (24.7)	91 (40.3)	123 (25.8)	
*APOE* ε4 − ^d^	4148 (70.0)	124 (54.9)	341 (71.6)	< 0.0001
**Lifetime history of depression**				
Yes	860 (14.5)	33 (14.6)	63 (13.2)	
No	5063 (85.4)	193 (85.4)	413 (86.8)	0.6225
**Lifetime history of diabetes**				
Yes	822 (13.9)	47 (20.8)	66 (13.9)	
No	4971 (83.9)	176 (77.9)	407 (86.5)	0.0174
**Lifetime history of cardiovascular disease**				
Yes	456 (7.7)	29 (12.8)	28 (5.9)	
No	5352 (90.3)	193 (85.4)	440 (92.4)	0.0016
**Subjective cognitive complaints**				
No	1688 (28.5)	36 (15.9)	115 (24.2)	
Occasional	3501 (59.1)	142 (62.8)	303 (63.7)	
Persistent	737 (12.4)	48 (21.2)	58 (12.2)	0.0014
	**Mean (SD)**	**Mean (SD)**	**Mean (SD)**	
**Age**	61.6 (6.5)	66.9 (5.1)	61.2 (6.5)	< 0.0001
**GFAP**	n.a	132.5 (79.7)	86.7 (46.8)	< 0.0001
**NFL**	n.a	22.0 (11.9)	15.7 (8.3)	< 0.0001
**p-tau181**	n.a	2.1 (1.2)	1.7 (1.2)	< 0.0001

Percentages might not sum up to 100 because of rounding and missing values

*GFAP* glial fibrillary acidic protein, *NfL* neurofilament light chain, *p-tau181* phosphorylated tau181, *SD* standard deviation

^a^Cases and controls from the nested case–control study embedded within the ESTHER cohort

^b^*p*-values derived from *t*-test tests for continuous and chi-square tests for categorical variables

^c^*APOE* ε4 + ; participants carrying the *APOE* ε4 allele (ε2ε4, ε3ε4, ε4ε4)

^d^*APOE* ε4 − ; participants not carrying the *APOE* ε4 allele (ε2ε2, ε3ε2, ε3ε3)

### Memory clinic-based sample

In the memory clinic sample, out of *n* = 430 patients, *n* = 422 had complete information relating to SCC, thereof *n* = 203 (48%) were women. The mean age was 74.9 years (SD 7.5). Dementia was diagnosed among 122 (29%) participants, and the most common forms were Alzheimer’s disease (*n* = 46; 11%) and mixed dementia (*n* = 33; 8%). SCD was diagnosed among *n* = 46 (11%) and depression among 180 (43%) patients. There were *n* = 28 patients with an uncertain diagnosis of neurodegenerative disorder, and they were excluded from the statistical analyses. Hence, the final sample consisted of *n* = 394 patients with *n* = 122 (31%) participants with dementia and *n* = 129 (33%) participants with a diagnosis of depression in the absence of dementia (Additional file 1: Table S1). Among participants with dementia, *n* = 38 had comorbidity of dementia and depression.

### Outcome all-cause dementia

Participants with persistent SCC had twofold increased odds of developing all-cause dementia over the 17-year follow-up (OR 2.15; 95%CI 1.52–3.06; *p*-value < 0.0001), but if participants with persistent SCC were also *APOE* ε4 carriers (*APOE* ε4 +), the odds increased to 5.35 (95%CI 3.25–8.81, *p*-value < 0.0001; Table 2).

**Table 2:** Longitudinal association of subjective cognitive complaints and *APOE* ε4 genotype with risk of all-cause dementia over 17-year follow-up (ESTHER cohort)

	**Incident dementia (*n* = 451), *N* (%)**	**No incident dementia (*n* = 5475), *N* (%)**	**Model 1**^**a**^** odds ratio (95%CI), *p*-value**^**c**^	**Model 2**^**b**^** odds ratio (95%CI), *p*-value**^**c**^
**Subjective cognitive complaints**				
No	81 (17.9)	1607 (29.4)	Reference	Reference
Occasional	270 (59.9)	3231 (59.0)	1.33 (1.02–1.74), 0.0373	1.32 (1.00–1.76), 0.0546
Persistent	100 (22.2)	637 (11.6)	2.18 (1.58–3.02), < 0.0001	2.15 (1.52–3.06), < 0.0001
***APOE* ε4**				
*APOE* ε4 − ^d^	254 (56.3)	3894 (71.1)	Reference	Reference
*APOE* ε4 + ^e^	170 (37.7)	1296 (23.7)	2.21 (1.78–2.74), < 0.0001	2.06 (1.65–2.57), < 0.0001
**Subjective cognitive complaints and *APOE*ε4**				
No and *APOE* ε4 −	46 (10.2)	1136 (20.7)	Reference	Reference
No and *APOE* ε4 +	32 (7.1)	388 (7.1)	2.34 (1.43–3.83), 0.0007	2.46 (1.49–4.08), 0.0005
Occasional and *APOE* ε4 −	158 (35.0)	2290 (41.8)	1.41 (0.99–2.00), 0.0598	1.49 (1.03–2.14), 0.0340
Occasional and *APOE* ε4 +	93 (20.6)	760 (13.9)	2.71 (1.85–3.99), < 0.0001	2.69 (1.80–4.01), < 0.0001
Persistent and *APOE* ε4 −	50 (11.8)	468 (8.5)	1.90 (1.22–2.95), 0.0043	2.14 (1.36–3.37), 0.0010
Persistent and *APOE* ε4 +	45 (11.1)	148 (2.7)	5.60 (3.47–9.03), < 0.0001	5.35 (3.25–8.81), < 0.0001

Percentages might not sum up to 100 because of rounding and missing values

*CI* confidence interval

^a^Logistic regression model adjusted for age (continuous), sex, and educational level

^b^Logistic regression model additionally adjusted for lifetime history of stroke, myocardial infarction, diabetes, lifetime history of depression, and *APOE* ε4 genotype (exception: subgroup analyses including *APOE* ε4 genotype)

^c^*p*-value derived from the logistic regression models

^d^*APOE* ε4 + ; participants carrying the *APOE* ε4 allele (ε2ε4, ε3ε4, ε4ε4)

^e^*APOE* ε4 − ; participants not carrying the *APOE* ε4 allele (ε2ε2, ε3ε2, ε3ε3)

The results of the ESTHER study relating to the association of persistent SCC with dementia were comparable to those found in the memory clinic sample (OR 1.66; 95%CI 1.06–2.60, *p*-value 0.0257; Additional file 1: Table S2), especially if in the clinical sample participants with depression were additionally excluded (OR 2.45; 95%CI 1.38–4.34; results not shown).

The mean values of all markers of neurodegenerative diseases were higher among dementia cases than controls, particularly for GFAP (Table 1), and the combination of persistent SCC with high GFAP levels yielded an approximatively eightfold increased risk of all-cause dementia (OR 7.52; 95%CI 2.79–20.29; *p*-value < 0.0001, Table 3). Associations of NfL and p-tau181 with the risk of dementia were not statistically significant either alone or in combination with SCC.

**Table 3:** Longitudinal association of biomarkers of neurodegenerative diseases and subjective cognitive complaints with risk of all-cause dementia (ESTHER cohort, nested case–control study)

	**Cases (*n* = 226), *N* (%)**	**Controls, (*n* = 476), *N* (%)**	**Model 1**^**a**^** odds ratio (95%CI), *p*-value**^**c**^	**Model 2**^**b**^** odds ratio (95%CI), *p*-value**^**c**^
**Subjective cognitive complaints**				
No	36 (15.9)	115 (24.2)	Reference	Reference
Occasional	142 (62.8)	303 (63.7)	1.22 (0.75–1.96), 0.4220	1.26 (0.76–2.08), 0.3748
Persistent	48 (21.2)	58 (12.2)	1.70 (0.93–3.12), 0.0874	1.58 (0.82–3.05), 0.1684
**GFAP**				
GFAP_Q1–3_	117 (51.8)	403 (84.7)	Reference	Reference
GFAP_Q4_	109 (48.2)	70 (14.7)	2.93 (1.94–4.41), < 0.0001	3.17 (2.03–4.95), < 0.0001
**Subjective cognitive complaints and GFAP**				
No and GFAP_Q1–3_	22 (9.7)	104 (21.8)	Reference	Reference
No and GFAP_Q4_	14 (6.2)	10 (2.1)	4.06 (1.41–11.68), 0.0093	3.98 (1.27–12.48), 0.0180
Occasional and GFAP_Q1–3_	76 (33.6)	251 (52.7)	1.30 (0.74–2.29), 0.3575	1.31 (0.73–2.38), 0.3674
Occasional and GFAP_Q4_	66 (29.2)	51 (10.7)	2.81 (1.47–5.38), 0.0017	3.02 (1.51–6.01), 0.0017
Persistent and GFAP_Q1–3_	19 (8.4)	48 (10.1)	1.22 (0.57–2.63), 0.6138	0.99 (0.43–2.30), 0.9809
Persistent and GFAP_Q4_	29 (12.8)	9 (1.9)	7.39 (2.89–18.88), < 0.0001	7.52 (2.79–20.29), < 0.0001
**NfL**				
NfL_Q1–3_	136 (60.2)	388 (81.5)	Reference	Reference
NfL_Q4_	90 (39.8)	85 (17.9)	1.32 (0.87–2.00), 0.1926	1.38 (0.88–2.17), 0.1573
**Subjective cognitive complaints and NfL**				
No and NfL_Q1–3_	22 (9.7)	96 (20.2)	Reference	Reference
No and NfL_Q4_	14 (6.2)	18 (3.8)	1.35 (0.53–3.41), 0.5273	1.52 (0.57–4.07), 0.4025
Occasional and NfL_Q1–3_	83 (36.7)	249 (52.3)	1.18 (0.66–2.10), 0.5737	1.25 (0.68–2.31), 0.4662
Occasional and NfL_Q4_	59 (26.1)	53 (11.1)	1.77 (0.91–3.46), 0.0950	1.95 (0.96–3.99), 0.0664
Persistent and NfL_Q1–3_	31 (13.7)	43 (9.0)	2.05 (0.99–4.25), 0.0547	2.00 (0.91–4.42), 0.0851
Persistent and NfL_Q4_	17 (7.5)	14 (2.9)	1.70 (0.66–4.36), 0.2689	1.63 (0.60–4.44), 0.3385
**p-tau181**				
p-tau181_Q1–3_	152 (67.3)	386 (81.1)	Reference	Reference
p-tau181_Q4_	74 (32.7)	90 (18.9)	1.20 (0.80–1.82), 0.3809	1.21 (0.78–1.89), 0.3936
**Subjective cognitive complaints and p-tau181**				
No and p-tau181_Q1–3_	24 (10.6)	93 (19.5)	Reference	Reference
No and p-tau181_Q4_	12 (5.3)	22 (4.6)	1.07 (0.42–2.72), 0.8875	1.00 (0.37–2.69), 0.9959
Occasional and p-tau181_Q1–3_	97 (42.9)	248 (52.1)	1.18 (0.67–2.07), 0.5685	1.18 (0.65–2.13), 0.5909
Occasional and p-tau181_Q4_	45 (19.9)	55 (11.6)	1.46 (0.74–2.87), 0.2749	1.55 (0.76–3.19), 0.2308
Persistent and p-tau181_Q1–3_	31 (13.7)	45 (9.5)	1.63 (0.79–3.34), 0.1854	1.53 (0.71–3.31), 0.2796
Persistent and p-tau181_Q4_	17 (7.5)	13 (2.7)	2.07 (0.79–5.43), 0.1413	1.73 (0.62–4.81), 0.2966

Percentages might not sum up to 100 because of rounding and missing values

In this sample GFAP_Q4_ ≥ 122.00 pg/mL; NfL_Q4_ ≥ 21.20 pg/mL; p-tau181_Q4_ ≥ 2.06 pg/mL

*CI* confidence interval, *GFAP* glial fibrillary acidic protein, *NfL* neurofilament light chain, *p-tau181* phosphorylated tau181, *Q* quartile

^a^Logistic regression model 1 adjusted for age (continuous), sex, and educational level

^b^Logistic regression model 2 additionally adjusted for lifetime history of stroke, myocardial infarction, diabetes, lifetime history of depression, and *APOE* ε4 genotype

^c^*p*-value derived from the logistic regression models

In these models, the biomarkers were tested separately. A (not shown) regression model including a combination of all biomarkers and assessing the association of such combination with dementia risk did not add informative value to the model including the combination of persistent SCC with high GFAP values (OR 2.40; 95%CI 1.16–4.96, *p*-value 0.0178). Similarly, persistent SCC alone did not add informative value in this model (OR 1.73; 95%CI 0.94–3.19, *p*-value 0.0791). Hence, these additional analyses supported the results showing that in this community-based cohort, high values of GFAP along with persistent SCC were the strongest predictor for dementia risk.

### Outcome depression and SCC

In the ESTHER cohort, the risk estimate of persistent SCC for the risk of the first depressive episode among participants without dementia was similar to that observed for dementia (OR 2.00; 95%CI 1.41–2.84, *p*-value 0.0001). In the memory clinic cohort, persistent SCC was associated with a threefold increased risk of depression in the absence of dementia (OR 2.94; 95%CI 1.77–488, *p*-value < 0.0001), and sensitivity analysis performed in a reduced sample only including cognitively unimpaired participants (*n* = 103) revealed an even stronger association between persistent SCC and diagnosis of depression (OR 7.10; 95%CI 2.76–18.26, *p*-value < 0.0001).

In the ESTHER cohort, the mean levels of biomarkers were comparable among individuals with and without incident depression. Levels of GFAP were higher among individuals without incident depression than among those with depression (88.5 pg/mL and 83.1 pg/mL, respectively), NfL levels were similar among the two groups (15.9 pg/mL and 15.5 pg/mL, respectively), and p-tau181 levels were slightly higher among depression cases (1.1 pg/mL) than among individuals without depression (0.95 pg/mL). Neither *APOE* ε4 nor any of the biomarkers were statistically significantly associated with incident depression (Table 4). Hence, for the outcome of depression, subjective complaints were not combined with markers of neurodegenerative diseases.

**Table 4:** Longitudinal association of subjective cognitive complaints, *APOE* ε4 genotype, and blood biomarkers with risk of depression over 17-year follow-up among participants without dementia (ESTHER cohort)

	**Incident depression (*n* = 505), *N* (%)**	**No incident depression (*n* = 4065), *N* (%)**	**Model 1**^**a**^** odds ratio (95%CI), *p*-value**^**c**^	**Model 2**^**b**^** odds ratio (95%CI), *p*-value**^**c**^
**Subjective cognitive complaints**				
No	131 (25.9)	1310 (32.2)	Reference	Reference
Occasional	309 (61.2)	2373 (58.4)	1.41 (1.13–1.76), 0.0022	1.43 (1.15–1.80), 0.0025
Persistent	65 (12.9)	382 (9.4)	2.05 (1.46–2.87), < 0.0001	2.00 (1.41–2.84), 0.0001
***APOE* ε4**				
*APOE* ε4 − ^d^	349 (69.1)	2885 (71.0)	Reference	Reference
*APOE* ε4^e^	130 (25.7)	960 (23.6)	1.06 (0.85–1.32), 0.5960	1.07 (0.85–1.33), 0.5800
**Subgroup with biomarker measurements**	*n* = 44	*n* = 360		
**GFAP**				
GFAP_Q1–3_	35 (79.6)	263 (73.1)	Reference	Reference
GFAP_Q4_	9 (20.4)	95 (26.4)	0.66 (0.29–1.51), 0.3225	0.68 (0.28–1.63), 0.3828
**NfL chain**				
NfL_Q1–3_	37 (84.1)	263 (73.5)	Reference	Reference
NfL_Q4_	7 (15.9)	95 (26.5)	0.50 (0.20–1.26), 0.1408	0.51 (0.20–1.34), 0.1737
**p-tau181**				
p-tau181_Q1–3_	30 (68.2)	273 (75.8)	Reference	Reference
p-tau181_Q4_	14 (31.8)	87 (24.2)	1.45 (0.72–2.96), 0.3020	1.61 (0.77–3.37), 0.2041

Percentages might not sum up to 100 because of rounding and missing values

In this sample GFAP_Q4_ ≥ 109.0 pg/mL; NfL_Q4_ ≥ 18.90 pg/mL; p-tau181_Q4_ ≥ 1.96 pg/mL

*CI* confidence interval, *GFAP* glial fibrillary acidic protein, *NfL* neurofilament light chain, *p-tau181* phosphorylated tau181, *Q* quartile

^a^Logistic regression model 1 adjusted for age (continuous), sex, and educational level

^b^Logistic regression model 2 additionally adjusted for lifetime history of stroke, myocardial infarction, diabetes, and *APOE* ε4 genotype (exception: subgroup analyses including *APOE* ε4 genotype)

^c^*p*-value derived from the logistic regression models

^d^*APOE* ε4 + ; participants carrying the *APOE* ε4 allele (ε2ε4, ε3ε4, ε4ε4)

^e^*APOE* ε4 − ; participants not carrying the *APOE* ε4 allele (ε2ε2, ε3ε2, ε3ε3)

## Discussion

This study showed that risk estimates of SCC for all-cause dementia and for depression in the absence of dementia were comparable, independently of the diagnostic setting and, as such, of little informative value. However, longitudinal analyses performed with the community-based cohort showed that the combination of SCC with *APOE* ε4 and GFAP could have the potential to contribute to the differentiation between future risk of all-cause dementia and risk of depression in the absence of dementia. Clinical data further indicated that among cognitively unimpaired individuals, persistent SCC alone might be more strongly associated with depression than with dementia.

### Outcome all-cause dementia

The prevalence of SCC in the ESTHER cohort was similar to that found in other large cohorts [12, 13], and the magnitude of the estimates for risk of dementia was comparable to that found in the memory clinic cohort and in a meta-analysis of longitudinal studies including more than 74,000 participants [23]. As supported by previous literature focusing on SCD, combining *APOE*ε4 + with SCC led to substantially increased odds of incident dementia as compared to those associated with SCC alone or *APOE* ε4 + alone [24]. Studies analyzing the associations of SCD with brain biomarkers for Alzheimer’s disease indicated that a notable proportion of participants with SCD fall within the Alzheimer’s disease continuum and are at increased risk of objective cognitive decline [7–9]. Emerging evidence from studies with blood biomarkers seems to indicate that GFAP might be a strong biomarker candidate for objective cognitive decline among individuals with SCD or cognitively normal older people [11, 25, 26]. This study additionally showed that the odds of GFAP for all-cause dementia are highly increased by combining high levels of GFAP with persistent SCC and that GFAP is able to predict the risk of all-cause dementia but not of depression in the absence of dementia.

The results of the regression models point to the superior diagnostic value of GFAP for dementia as compared to SCC alone and to the other biomarkers of neurodegenerative disease. The lack of additional diagnostic value of SCC in a model including a combination of all markers also indicated the superiority of the biological information (GFAP) compared to the subjective (SCC) one, but all analyses supported the results indicating that the predictive value of GFAP for dementia was greatly enhanced by the addition of subjective information.

The weak associations of NfL and p-tau181 with all-cause dementia and the results obtained with GFAP, a marker of reactive astrocytes, shall be interpreted in relation to the nature of the ESTHER cohort, which includes participants with mixed pathology, irrespective of the reported dementia diagnosis, as reported in detail in previous work [20]. It is in fact known that in the presence of brain injury, including vascular and neurodegenerative injury, astrocytes undergo structural, molecular, and functional changes and also undergo a transition into their reactive phenotype [27]. Reactive astrocytes lose their ability to regulate adult neurogenesis [28] and to control circuits involved in learning and memory [29], which accelerate cognitive decline, especially among individuals with a diffuse cerebrovascular injury who might be more subject to widespread astrogliosis than individuals with localized brain lesions. These biological processes might contribute to explaining why GFAP is the strongest risk marker for clinical diagnosis of dementia among ESTHER participants with a high prevalence of mixed pathology and why GFAP plays a key role for all-cause dementia but not for the risk of depression in the absence of dementia.

### Outcome depression

In the ESTHER cohort, none of the biomarkers was associated with the risk of the first depressive episode in the absence of dementia, but the estimates for p-tau181 seemed to show an increased risk for depression. Depression with cognitive impairment in elderly persons and Alzheimer's disease seems to share common biological mechanisms [30]. During a long follow-up period, as was the case in the ESTHER cohort, the co-occurrence of cognitive and mood symptoms in certain individuals could explain the observed increase of pTau181. Hence, future studies with higher statistical power might reveal a relationship between p-tau181 and the risk of depression among older people.

Recent studies found higher GFAP levels to be associated with depressive symptoms [31, 32] and neuroticism [33]. These studies were cross-sectional and reflect the role of inflammation in the pathophysiology of depression. However, they cannot be compared with our findings because the ESTHER study is a prospective cohort, and GFAP measurements were performed in baseline blood taken at recruitment, years before diagnoses of dementia and depression were made. Furthermore, these analyses are limited to the occurrence of the first depressive episode among older people. As such, our findings expand these previous studies by elucidating the value of GFAP for differential risk assessment of all-cause dementia and depression and by adding the information relating to SCC and *APOE* ε4.

Both the results based on the community and memory clinic cohort clearly showed that the information relating to SCC alone could not discriminate between patients with depression in the absence of dementia and patients with dementia, supporting the concept of SCC as unspecific and transdiagnostic both in community- and clinic-based settings. Interestingly, the strongest association was found between persistent SCC and depression among cognitively unimpaired individuals. This result seems to show that SCC in clinical settings might have a greater informative value for a diagnosis of depression than for a diagnosis of cognitive deterioration due to neurodegenerative disorders. Unfortunately, we could not repeat such analyses in the community sample, because in this sample we do not have information on cognitive impairment not amounting to dementia, but if such results are repeated in better-characterized community cohorts, they could provide relevant guidance for GPs confronting with SCC among older patients.

### Strengths and limitations

One of the main strengths of this study is the representativeness of the study population, whose distribution of baseline characteristics was similar to the distribution in the respective age categories of a representative sample of the German population assessed through the German National Health Survey [18]. Further strengths are the long follow-up time; the focus on individuals with mixed pathology, which is a common medical condition scarcely explored in association with blood biomarkers of neurodegenerative diseases; and the longitudinal analyses, which allowed to disentangle the risk of developing dementia from the risk of developing depression in the absence of dementia. The lack of a standardized dementia and cognitive assessment at baseline and follow-up is a major limitation of the ESTHER cohort and of this study. A further limitation is the potential attrition bias since participants with cognitive deficits might have been lost to follow-up. Due to the small sample size including measurements of blood biomarkers, biomarkers could not be combined with genetic information. The small samples also impose caution in interpreting the results, especially those relating to subgroup analyses. Another note of caution is that diagnoses of depression were made differently in the two cohorts and that the results of the ESTHER study can only be generalized to the older German population.

## Conclusions

In conclusion, this study suggests that in community settings, a few simple cognitive questions can reveal the risk of several medical conditions and that the addition of biological information, especially GFAP and *APOE* ε4, could add specificity and has the potential to be used as a screening instrument in primary care for differentiating between risk assessment of future dementia and depression.

### Supplementary Information

**Additional file 1: Table S1.** Basic characteristics of the memory-clinic participants. **Table S2.** Cross-sectional associations of subjective cognitive complaints with diagnosis of dementia and depression (clinic-based data).

## Data Availability

Due to restrictions of informed consent, the data cannot be made publicly available, but they are available upon reasonable request with appropriate proposal and approval from the principal investigators of the cohort studies.

## Abbreviations

AD: Alzheimer’s dementia
*APOE **ε4*: Apolipoprotein E ε4
CI: Confidence interval
GDS: Geriatric Depression Scale
GFAP: Glial fibrillary acidic protein
GP: General practitioner
MCI: Mild cognitive impairment
MMSE: Mini-Mental State Examination
NfL: Neurofilament light chain
OR: Odds ratio
p-tau181: Phosphorylated tau181
SCC: Subjective cognitive complaints
SCD: Subjective cognitive decline
SD: Standard deviation
