# Supplementary material for: Subjective cognitive complaints and blood biomarkers of neurodegenerative diseases: a longitudinal cohort study
Source: Alzheimers Res Ther. 2023 Nov 11;15:198. doi: 10.1186/s13195-023-01341-3 (PMC10638700; doi:10.1186/s13195-023-01341-3)
Supplement: Supplementary file 1 — Additional file 1: Table S1. Basic characteristics of the memory-clinic participants. Table S2. Cross-sectional associations of subjective cognitive complaints with diagnosis of dementia and depression (clinic-based data). [file 13195_2023_1341_MOESM1_ESM.docx]

**Supplementary Table 1** Basic characteristics of the memory-clinic participants

|  |  | **Dementia** | |  | **Depression among participants without dementia** | |
| --- | --- | --- | --- | --- | --- | --- |
|  |  | yes=122  n (%) | no=272  n (%) |  | yes=129  n (%) | no=143  n (%) |
| **Sex** |  |  |  |  |  |  |
| female |  | 49 (40.2) | 138 (50.7) |  | 70 (54.3) | 68 (47.5) |
| male |  | 73 (59.8) | 134 (49.3) |  | 59 (45.7) | 75 (52.5) |
|  |  |  |  |  |  |  |
| **Age** |  |  |  |  |  |  |
| ≤ 70 |  | 26 (21.3) | 92 (33.8) |  | 51 (39.5) | 41 (28.7) |
| > 70 |  | 96 (78.7) | 180 (66.2) |  | 78 (60.5) | 102 (71.3) |
|  |  |  |  |  |  |  |
| **Education** |  |  |  |  |  |  |
| ≤ 9 years |  | 58 (47.5) | 163 (59.9) |  | 74 (57.4) | 89 (62.2) |
| 10-11 years |  | 36 (29.5) | 56 (20.6) |  | 25 (19.4) | 31 (21.7) |
| ≥ 12 years |  | 26 (21.3) | 52 (19.1) |  | 30 (23.2) | 22 (15.4) |
|  |  |  |  |  |  |  |
| **Subjective cognitive complaints** |  |  |  |  |  |  |
|  |  |  |  |  |  |  |
| no |  | 5 (4.1) | 19 (7.0) |  | 4 (3.1) | 15 (10.5) |
| occasional |  | 46 (37.7) | 133 (48.9) |  | 51 (39.5) | 82 (57.3) |
| persistent |  | 71 (58.2) | 120 (44.1) |  | 74 (57.4) | 46 (32.2) |
|  |  |  |  |  |  |  |
| **MMSE – total score** |  |  |  |  |  |  |
|  |  | **Mean (SD)** | **Mean (SD)** |  | **Mean (SD)** | **Mean (SD)** |
|  |  | 20.9 (5.4) | 26.6 (3.3) |  | 26.2 (3.5) | 27.0 (3.0) |
| **CERAD – total score** |  |  |  |  |  |  |
|  |  | **Mean (SD)** | **Mean (SD)** |  | **Mean (SD)** | **Mean (SD)** |
|  |  | 51.0 (25.0) | 71.4 (20.7) |  | 67.5 (25.5) | 74.9 (14.3) |
| **GDS – total score** |  |  |  |  |  |  |
|  |  | **Mean (SD)** | **Mean (SD)** |  | **Mean (SD)** | **Mean (SD)** |
|  |  | 3.3 (2.6) | 3.2 (2.9) |  | 4.9 (3.2) | 1.9 (1.7) |
|  |  |  |  |  |  |  |

MMSE, Mini Mental State Examination; CERAD, Consortium to Establish a Registry for Alzheimer’s Disease; GDS, Geriatric Depression Scale; SD, standard deviation.

**Supplementary Table 2** Cross-sectional associations of subjective cognitive complaints with diagnosis of dementia and depression (clinic-based data)

|  |  | **Dementia** | | |  | **Depression among participants without dementia** | | |
| --- | --- | --- | --- | --- | --- | --- | --- | --- |
|  |  | **yes=122**  n (%) | **no=272**  n (%) | **odds ratio^a)^**  (95%CI)  p-value |  | **yes=129**  n (%) | **no=143**  n (%) | **odds ratio^a)^**  (95%CI)  p-value |
| **Subjective cognitive complaints** |  |  |  |  |  |  |  |  |
|  |  |  |  |  |  |  |  |  |
| no/occasional |  | 51 (41.8) | 152 (55.9) | reference |  | 55 (42.6) | 97 (67.8) | reference |
| persistent |  | 71 (58.2) | 120 (44.1) | 1.66 (1.06-2.60)  0.0257 |  | 74 (57.4) | 46 (32.2) | 2.94 (1.77-4.88)  <0.0001 |
|  |  |  |  |  |  |  |  |  |

CI, confidence interval

1. Logistic regression models adjusted for age (continuous), sex, and educational years (continuous).
